# Supplementary material for: The interactome of intact mitochondria by cross-linking mass spectrometry provides evidence for coexisting respiratory supercomplexes
Source: Mol Cell Proteomics. 2017 Dec 8;17(2):216–32. doi: 10.1074/mcp.RA117.000470 (PMC5795388; doi:10.1074/mcp.RA117.000470)
Supplement: Supplemental Data [file supp_RA117.000470_133922_0_supp_23978_fzffwf.zip › spectra_annotation/mito_spectra_annotation/42-2-9-1-1-1.pdf]

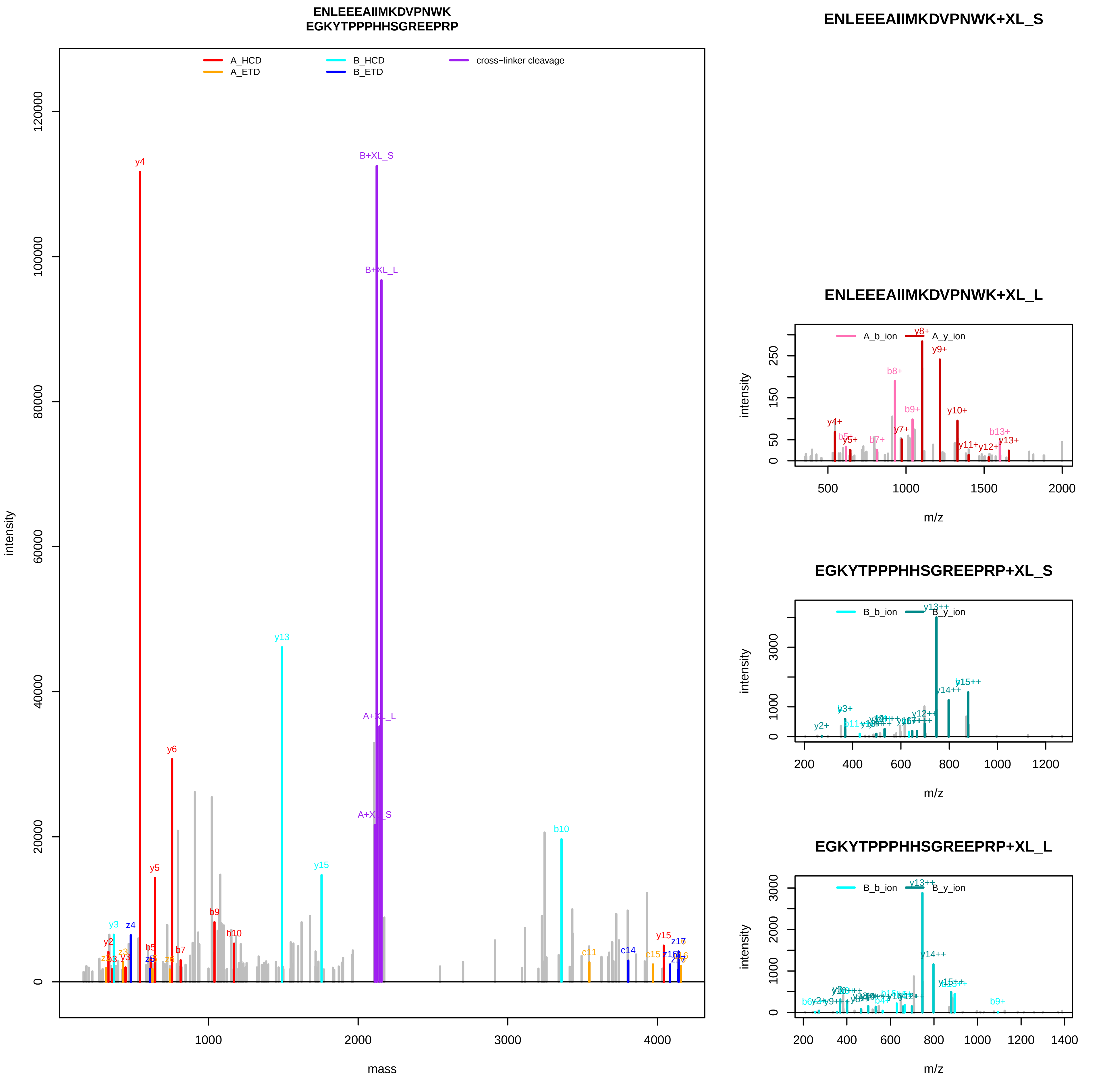
ENLEEEAIIMKDVPNWK
EGKYTPPPHHSGREEPRP
A_HCD
A_ETD
B_HCD
B_ETD
cross−linker cleavage
intensity
mass
B+XL_S
B+XL_L
A+XL_L
A+XL_S
y4
y6
y5
y13
y15
b10
b9
y3
z4
y2
b5
b7
y15
c11
c14
c15
z17
ENLEEEAIIMKDVPNWK+XL_S
ENLEEEAIIMKDVPNWK+XL_L
A_b_ion
A_y_ion
y8+
y9+
b8+
b9+
y10+
y4+
y7+
y5+
b7+
y11+
y12+
b13+
y13+
m/z
EGKYTPPPHHSGREEPRP+XL_S
B_b_ion
B_y_ion
y13++
y15++
y14++
y12++
y3+
y2+
EGKYTPPPHHSGREEPRP+XL_L
b9+
